# Drug-related problems in patients admitted for SARS-CoV-2 infection during the COVID-19 pandemic

**DOI:** 10.3389/fphar.2022.993158

**Published:** 2022-11-24

**Authors:** J. Barceló-Vidal, D. Echeverría-Esnal, N. Carballo, M. De Antonio-Cuscó, X. Fernández-Sala, M. E. Navarrete-Rouco, E. Colominas-González, S. Luque, M. Fuster-Esteva, L. Domingo, M. Sala, X. Duran, S. Grau, O. Ferrández

**Affiliations:** ^1^ Pharmacy Department, Hospital del Mar, Parc de Salut Mar, Barcelona, Spain; ^2^ Institut Hospital del Mar d’Investigacions Mèdiques (IMIM), Barcelona, Spain; ^3^ Faculty of Medicine, Universitat Pompeu Fabra (UPF), Barcelona, Spain; ^4^ Department of Epidemiology and Evaluation, Barcelona, Spain; ^5^ Statistics Deparment, Institut Hospital del Mar D'Investigacions Mèdiques (IMIM), Barcelona, Spain

**Keywords:** drug-related problem (DRP), COVID-19, SARS-CoV-2, pharmaceutical care, antiinfective agents, medication related problem, medication error, drug medication safety

## Abstract

**Introduction:** Drug-related problems (DRP) are events or circumstances in which drug therapy does or could interfere with desired health outcomes. In December 2019, a new coronavirus, SARS-CoV-2, appeared. Little knowledge about this type of infection resulted in the administration of various drugs with limited use in other pathologies. Evidence about DRP in patients with COVID-19 is lacking.

**Objective:** The aim of the present study is to describe identified cases of DRP and those drugs involved in the first wave of patients with COVID-19, and evaluate associated risk factors.

**Material and methods:** Observational, retrospective study performed in a tertiary university hospital between 14th March 2020 and 31 May 2020 (corresponding to the first COVID-19 wave). We recruited patients admitted during the study period. Exclusion criteria included age < 18 years; admission to critically ill units; and care received either in the emergency room, at-home hospitalization or a healthcare center.

**Results:** A total of 817 patients were included. The mean age was 62.5 years (SD 16.4) (range 18–97), and 453 (55.4%) were male. A total of 516 DRP were detected. Among the patients, 271 (33.2%) presented at least one DRP. The mean DRP per patient with an identified case was 1.9. The prevailing DRPs among those observed were: incorrect dosage (over or underdosage) in 145 patients (28.2%); wrong drug combination in 131 (25.5%); prescriptions not in adherence to the then COVID-19 treatment protocol in 73 (14.1%); prescription errors due to the wrong use of the computerized physician order entry in 47 (9.2%); and incorrect dosage due to renal function in 36 (7%). The logistic regression analysis showed that patients who received only prescriptions of antibacterials for systemic use (J01 ATC group) faced a higher likelihood of experiencing a DRP (OR 2.408 (1.071–5.411), *p* = 0.033).

**Conclusion:** We identified several factors associated with an increased risk of DRPs, similar to those reported in other pre-pandemic studies, including a prolonged length of stay, higher number of prescribed drugs and antimicrobial administration. The relevance of pharmacists and tools like pharmacy warning systems can help prevent, identify and resolve DRP efficiently.

## Introduction

Drug-related Problems (DRP) are events or circumstances in which drug therapy does or could interfere with desired health outcomes (The PCNE Classification for drug-related problems V9.01). According to current literature, the rate of DRP in hospitalized patients varies highly, ranging from 10% to 81.3% (Ferrández et al., 2019) (Bedouch et al., 2009) (Roten et al., 2010) (Blix et al., 2006).

Some factors associated to DRP developing such as older age, a higher comorbidity index or a higher number of concomitant drugs have been described (Urbina et al., 2014) (Alhawassi et al., 2014). Also, the utilization of some specific pharmacological groups such as opioids, diuretics, anticoagulants, antimicrobials and drugs of the cardiovascular system in general have frequently been implicated in the development of DRP in admitted patients (Ferrández et al., 2018). In addition, DRP have been associated to higher morbidity and mortality in admitted patients (Classen et al., 1997) and have been associated with a longer hospital stay and increased cost of hospitalization. Thus, it is essential to adopt strategies with the aim of increasing safety in the process of drug use.

In December 2019, severe acute respiratory syndrome coronavirus 2 (SARS-CoV-2) appeared, being first reported in Wuhan, China. The pathogen is responsible for causing COVID-19, a disease capable of developing pneumonia in patients (WHO int, 2021a) (Huang et al., 2020). It was not until the number of cases reached an exceedingly high figure that the World Health Organization declared the health crisis as a pandemic (WHO int, 2021b). Little knowledge about the infection resulted in the use of different drugs, which had a limited use in other pathologies, including tocilizumab, sarilumab, hydroxychloroquine, chloroquine, steroids, and remdesivir (a novel, specific antiviral). Furthermore, the increased administration of these drugs, coupled with the low familiarity of such use, led to a continuous need for pharmacological-related updates, so as to minimize potential medication errors and elevate drug medication safety. Finally, during the first wave of the pandemic, it is important to emphasize the fact that physicians of different specialties attended patients with COVID-19. This may suggest that there was a higher probability to induce medical errors and DRP.

Currently, there is a lack of evidence regarding DRP in patients with COVID-19 (Perez et al., 2021) (Gourieux et al., 2021). The aim of the present study is to describe reported cases of DRP and drugs administered in patients with COVID-19 during the first wave, and evaluate associated risk factors.

## Materials and methods

### Study design

Observational retrospective study performed in a tertiary university hospital between 14th March 2020 and 31st May 2020 (corresponding to the first COVID-19 wave).

### Setting

The study took place in a tertiary university hospital that provides 431 beds (413 are conventional beds while 18 are for those patients with a critically ill status). During the pandemic, the hospital adapted a total of seven hospitalization units to exclusively care for patients with COVID-19. Additionally, the hospital repurposed surgical intensive care units and operating rooms for critically ill patients with COVID-19 (68 beds in total). Moreover, different hospital infrastructures underwent restructuring for wider coverage of patients with COVID-19, bringing the total number of available beds to 585.

### The patient population

We included those patients admitted for COVID-19 during the study period. Exclusion criteria included age < 18 years; admission to critically ill units; and care received either in the emergency room, at-home hospitalization or a healthcare center. Computerized physician order entry (CPOE) was operational for all hospital beds. CPOE incorporates a pharmacy warning system (PWS) that generates drug alerts based on demographic data, drug dosage, laboratory tests related to the prescribed drug (such as renal and liver function profile, coagulation and electrolytes) and drug combinations (interactions, duplications, and necessary combinations). CPOE and PWS structures have been described elsewhere (Ferrández et al., 2017). Clinical pharmacists reviewed medical prescriptions on a daily basis. When a potential DRP was detected, an annotation with a recommendation was made in the patient’s medical record.

### Data collection

We prospectively recorded the following patient variables: demographic (sex, age), Charlson Comorbidity Index; pathologies potentially related to DRP [obesity, cachexia, kidney and hepatic dysfunction, diabetes mellitus, chronic obstructive pulmonary disease (COPD), and congestive heart failure, among others]; number of drugs received during hospitalization; and DRP detected by the PWS and during daily reviews of medications. DRP were classified per Pharmaceutical Care Network Europe Classification V.9.01 (PCNE). Drugs were classified according to the Anatomical Therapeutic Chemical Classification System (ATC) (WHO Collaborating Center for Drug Statistics Methodology, 2011, The, 2021).

### Statistical analysis

Absolute and relatives frequencies were used for categorical variables, while mean, standard deviation, median, and 25th and 75th percentiles for quantitative variables. A bivariate analysis of the data was performed to determine the possible relationship between the presence of at least one DRP at admission and each of the variables analyzed. Either the Chi-squared test or Fisher’s exact test was used for categorical variables, when deemed appropriate. For quantitative variables, either the Student’s *t*-test was used for independent data or the non-parametric Mann-Whitney *U*-test.

The risk of presenting at least one DRP was modeled with the results obtained using a multivariate binary logistic regression model. Variables that presented a statistical significance lower than 0.1 in the bivariate analysis were introduced. The variables were eliminated from the model if exclusion did not significantly modify either the model’s plausibility or coefficients of the remaining variables. Subsequently, we obtained the final model, including the odds ratio and 95% confidence intervals for each of the resulting variables.

*p*-values lower than 0.05 were considered statistically significant. To calculate such values, we used SPSS 18.0 statistical package (IBM Corp. New York. United States).

### Ethics

This study was approved by an independent ethics committee (Comité de Ética de la Investigación con medicamentos del Parc de Salut Mar) (reference number 2020/9584). No additional informed consent was required.

## Results

**FIGURE 1:**
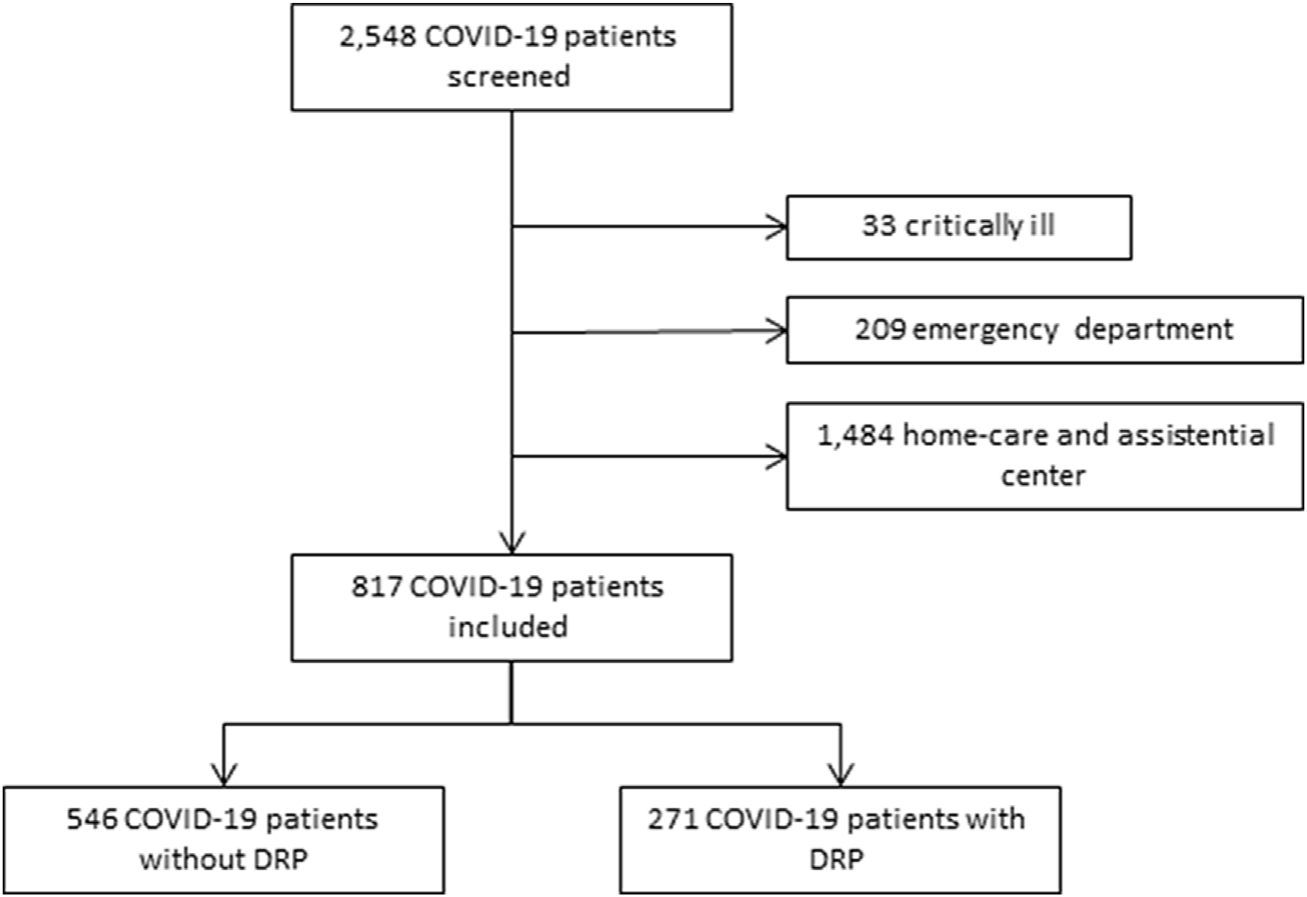
Study profile.

A total of 2,548 patients were recruited for the study. However, as Figure 1 details, 1,731 patients met exclusion criteria. The study, therefore, included a total of 817 patients. The mean age was 62.5 years (SD 16.4) (range 18–97), and 453 (55.4%) were males. Five hundred and sixteen DRP were reported. Of the patients, 271 (33.2%) presented at least one DRP. The mean DRP per patient with an identified case was 1.9.

The prevailing DRPs among those observed were: incorrect dosage (over or underdosage) in 145 patients (28.2%); wrong drug combination in 131 (25.5%); prescriptions not in adherence to the then COVID-19 treatment protocol in 73 (14.1%); prescription errors due to the wrong use of CPOE in 47 (9.2%); and incorrect dosage due to renal function in 36 (7%).

Table 1 shows results obtained from the bivariate analysis comparing hospitalized patients with the presence of any DRP and those without DRPs. In the group of patients who developed at least one DRP, the mean Charlson Comorbidity Index score was 3.4 (2.5) vs. 2.8 (2.5) (*p* < 0.001) in the group of patients who did not develop any DRP. This figure suggests that a higher prevalence of comorbidities is related to the development of a DRP. The mean length of stay was higher compared to the in-patient group without any DRP [17.3 (16.1) vs. 8.7 (5.6) days (*p* < 0.001)]. This was also observed with respect to the number of drugs administered during admission [15.1 (8.0) vs. 10.1 (4.3) drugs (*p* < 0.001)] and the prevalence of moderate-to-severe renal disease [51 (18.8%) vs. 42 (7.7%) patients (*p* < 0.001)] (Table 1).

**TABLE 1:** Bivariate analysis. Demographic and clinical characteristics of patients presenting at least one DRP vs. patients not presenting any DRP.

Variables	Hospitalizations without DRP, *n* = 546	Hospitalizations with DRP, *n* = 271	*p*-value
Gender			
Male	301 (55.1%)	152 (56.1%)	0.795
Female	245 (44.9%)	119 (43.9%)	
Age (years)			
Mean, DE	61.5 (16.8)	64.5 (15.4)	0.014
Median (P25-P75)	62 (50.0–75.0)	65 (54.0–77.0)	
Charlson comorbidity index			
Mean, DE	2.8 (2.5)	3.4 (2.5)	<0.001
Median (P25-P75)	2.0 (1.0–4.0)	3.0 (1.0–5.0)	
Length of stay			
Mean, DE	8.7 (5.6)	17.3 (16.1)	<0.001
Median (P25-P75)	7.0 (5.0–11.0)	13.0 (8.0–22.0)	
Number of drugs during stay			
Mean, DE	10.1 (4.3)	15.1 (8.0)	<0.001
Median (P25-P75)	9.0 (7.0–13.0)	13.0 (9.0–19.0)	
Obesity	56 (10.3%)	29 (10.7%)	0.845
Cachexia	2 (0.4%)	1 (0.4%)	0.995
Moderate-severe renal dysfunction	42 (7.7%)	51 (18.8%)	<0.001
Hepatic dysfunction	2 (0.4%)	3 (1.1%)	0.201
Deceased	2 (0.4%)	21 (7.7%)	0.829
ATC group prescribed			
A: alimentary tract and metabolism	439 (80.4%)	242 (89.3%)	0.001
B: blood and blood-forming metabolism	530 (97.1%)	267 (98.5%)	0.205
C: cardiovascular system	263 (48.2%)	173 (63.8%)	<0.001
D: dermatological	10 (1.8%)	11 (4.1%)	0.058
G: genitourinary system and sex hormones	71 (13.0%)	69 (25.5%)	<0.001
H: systemic hormonal preparations	215 (39.4%)	172 (63.5%)	<0.001
J: anti-infectives for systemic use	491 (89.9%)	262 (96.7%)	0.001
L: antineoplastic and immunomodulating agents	498 (91.2%)	245 (90.4%)	0.707
M: musculo-skeletal system	525 (96.2%)	260 (95.9%)	0.883
N: nervous system	260 (47.6%)	165 (60.9%)	<0.001
P: anti-parasitic products, insecticides and repellents	488 (89.4%)	236 (87.1%)	0.331
R: respiratory system	160 (29.3%)	124 (45.8%)	<0.001
S: sensory organs	22 (4.0%)	22 (8.1%)	0.015
V: various	15 (2.7%)	18 (6.6%)	0.008

DRP, Drug-related problems; SD, standard deviation; ATC, anatomical therapeutic chemical classification.

The top three most prescribed drugs in all admitted patients with COVID-19 were those from group B (blood and blood-forming organs) [797 (97.55%)], followed by group M (musculo-skeletal system) [785 (96.1%)] and, finally, group J (anti-infectives for systemic use) [753 (92.17%)] according to ATC classification. Of these, a significant difference was observed with prescriptions made from groups A (alimentary tract and metabolism), C (cardiovascular system), G (genitourinary system and sex hormones), H (systemic hormonal preparations), J (anti-infectives for systemic use), N (nervous system), and R (respiratory system).

Nevertheless, the logistic regression analysis (detailed logistical regression shown in Table 2) showed that only patients who received prescriptions of anti-infectives for systemic use (J01 ATC group) faced a higher likelihood of experiencing a DRP (OR 2.408 (1.071–5.411), *p* = 0.033). Moreover, this J01 ATC group was the main ATC group detected (124, 24.0%), especially when drug therapy involved ceftriaxone [68 (54.84%)] and azithromycin [36 (29.03%)] prescriptions—which was recommended in COVID-19 protocols at the time. The prevailing DRPs among those observed from J01 DRP were: incorrect dosage (over or underdosage) in 67 patients (54%); wrong drug combination in 18 (14.5%); prescriptions not in adherence to the then COVID-19 treatment protocol in 15 (12.1%); prescription errors due to the wrong use of CPOE in 11 (8.9%); DRP related with antimicrobial spectrum in 8 (6.4%); and incorrect dosage due to renal function in 5 (4%).

**TABLE 2:** Logistical regression. Patient variables associated with the presence of at least one DRP.

Logistic regression (crude)			Logistic regression adjusted by age	Logistic regression adjusted by length of stay	Logistic regression adjusted by number of drugs
Variable	OR (IC95%)	*p*	OR (IC95%)	OR (IC95%)	OR (IC95%)
Sex (females)	0.990 (0.703–1.396)	0.957	—	—	—
Charlson comorbidity Index	0.935 (0.836–1.045)	0.236	0.915 (0.839–0.998) (*p* = 0.046)	—	—
Length of stay (days)	1.071 (1.040–1.103)	<0.001	1.071 (1.040–1.103) (*p* < 0.001)	—	1.103 (1.074–1.133) (*p* < 0.001)
Number of drugs during stay	1.146 (1.075–1.222)	<0.001	1.149 (1.078–1.224) (*p* < 0.001)	1.231 (1.162–1.304) (*p* < 0.001)	—
Age	0.995 (0.979–1.012)	0.553	—	—	—
ATC group prescribed					
A: alimentary tract and metabolism	0.782 (0.464–1.317)	0.355	—	—	—
B: blood and blood-forming metabolism	1.046 (0.327–3.349)	0.940	—	—	—
C: cardiovascular system	0.924 (0.606–1.408)	0.712	—	—	—
D: dermatological	0.924 (0.283–3.021)	0.896	—	—	—
H: systemic hormonal preparations	1.099 (0.755–1.600)	0.622	—	—	—
J: anti-infectives for systemic use	1.983 (0.851–4.413)	0.115	—	—	2.408 (1.071–5.411) (*p* = 0.033)
L: antineoplastic and immunomodulating agents	1.008 (0.376–2.702)	0.987	—	—	—
M: musculo-skeletal system	0.444 (0.188–1.049)	0.064	—	0.419 (0.178–0.982) (*p* = 0.045)	—
N: nervous system	0.744 (0.499–1.108)	0.146	—	0.675 (0.456–0.998) (*p* = 0.049)	—
P: anti-parasitic products, insecticides and repellents	0.441 (0.185–1.053)	0.065	—	—	—
R: respiratory system	0.939 (0.645–1.366)	0.741	—	—	—
S: sensory organs	0.928 (0.429–2.007)	0.850	—	—	—

ATC, anatomical therapeutic chemical classification; OR, odds ratio; CI, confidence interval.

The degree of acceptance of all pharmaceutical interventions when a DRP was detected was 76.9%. In 7.4% of interventions, the DRP had already resolved before the result could be assessed. Among all pharmaceutical interventions, the degree of acceptance of pharmaceutical intervention was assessable in 478 cases. From these, 84.3% were accepted. Twenty-one (7.7%) patients with at least one DRP died while hospitalized, while 40 (7.3%) patients without any DRP died (*p* = 0.829).

## Discussion

The first wave of COVID-19 required clinicians and researchers alike to build their knowledge of this new pathology and its related treatment quickly. Although advances have been made, there are still some uncertainties about care in patients with COVID-19. In the present study, at least one DRP was detected in one-third of admissions, with incorrect dosage (over or underdosage) being the main DRP reported. In the logistic regression analysis, only hospital stay, the number of drugs and administration of antimicrobials of systemic use were associated with the presence of at least any DRP.

The number of DRP observed in this study is higher than in other reported series performed during the first wave of pandemic. In one study done in a French university hospital, 28.8% of patients with COVID-19 presented at least one DRP during a 1-month study period (Perez et al., 2021). Another study noted that 19.1% of patients with COVID-19 treated with lopinavir/ritonavir, hydroxychloroquine or azithromycin presented at least one DRP in relation to those drugs (Gourieux et al., 2021). The rate of DRP observed in patients with COVID-19 was higher than in other previous studies carried out in patients without COVID-19 (Ferrández et al., 2019) (Ferrández et al., 2018) (Krähenb et al., 2007). However, it is worth mentioning that the rate could have been affected by the age of those patients evaluated (Hailu et al., 2020).

Among all DRPs identified, incorrect dosage (over or underdosage) was the main one (28.1%). This was also the primary DRP reported in a study wherein the objective was to compare pharmaceutical interventions in patients with and without COVID-19 (27.9% vs. 47.6%, respectively) (Perez et al., 2020). However, a separate study observed that only 10.2% of the 59 pharmaceutical interventions performed in patients with COVID-19 treated with lopinavir/ritonavir, hydroxychloroquine or azithromycin were related with an incorrect dosage; wrong duration of treatment (54.2%) and the presence of interactions (23.7%) comprised the main DRPs (Gourieux et al., 2021).

Different pharmacological groups were associated with the onset of any DRP in the univariate analysis. However, in the multivariate analysis, when adjusted by the number of administered drugs, only antimicrobials of systemic use (J01) presented an association with any DRP. Antimicrobials of systemic use were present in 96.7% of patients with at least one DRP versus 89.9% of patients without any DRP. The former was one of the main pharmacological groups involved in the 188 (17.8%) DRP identified in one study evaluating patients with and without COVID-19 (Perez et al., 2021). Penicillin, followed by macrolides, were the most commonly identified antimicrobials. Also, in a study from an acute geriatric unit analyzing a total of 355 patients before and during the first COVID-19 wave, DRP were concluded to be mostly related to anti-infectious drugs during the pandemic (20.3%, *p* = 0.038) (M. Chappea, M. Corvaisier, A. Brangier, C. Annweiler, 2022).

One study did evaluate DRP in relation to antivirals administered for this type of infection (Gourieux et al., 2021). Nevertheless, in the present study, no DRP with specific COVID-19 antivirals such as remdesivir was identified. During the study period, this drug was not commercially available.

The study performed by Chappea et al., 2022, compared DRP identified prior to and during the first wave of COVID-19. Even they included less number of patients, this group found a higher proportion of pharmaceutical interventions during pandemic. In addition, interventions of anti-infective agents rose. Even though we did not compare our study population with patients admitted prior the first wave, our number of pharmaceutical interventions was higher than previous series, and DRP development was associated with anti-infective drugs prescription. On the other hand, they found a lower acceptance rate of the interventions than our study.

Our study also observed a longer hospital stay in those patients presenting at least one DRP. However, it is not known if this finding is related to a specific DRP or in association with the COVID-19 infection. There were no differences in mortality between groups.

This study did, however, present some limitations. For example, we did not include critically ill patients with COVID-19, which could have enlightened the kind of DRP and medication involved in DRP in these patients. However, a specific design would be necessary for this group of patients due to their individualized characteristics. Another limitation is the unawareness is the clinical impact associated to DRP. Additionally, as we did not include patients without COVID-19 from the study period patients during the study period, we could not perform a comparison between both groups.

Strengths of the study included a high number of patients and an extended study period—indeed, remarkably more than that reported in other studies. In addition, the comorbidities of the patients are well described, which allows a better understanding of the patient profile that is susceptible to suffering a DRP.

While the results obtained in this study may seem obsolete today, these insights may provide useful information with respect to new COVID-19 waves and the management of breakthrough drugs. In the same vein, the presence of novel drugs like nirmatrelvir/ritonavir (Paxlovid^®^), which has a high drug-drug interaction rate, underpins the importance of pharmacists in supporting decisions made about COVID-19 therapy and enhancing overall drug safety.

During the pandemic, the role of pharmacists and their importance became more clearly evident (Bragazzi et al., 2020) (Mallhi et al., 2020). Perhaps the lack of knowledge regarding COVID-19 and its evolution, as well as pharmacological management, has ushered in a new area to operate.

In conclusion, several factors were associated with a higher risk of a DRP, including prolonged length of stay, a higher number of prescribed drugs and administration of antimicrobials. This finding is similar to that reported in other pre-pandemic studies. However, the inclusion of drugs less known in COVID-19 therapeutic armamentarium—as well as high rotation of such drugs due availability—have underscored the relevance of pharmacists, especially as it relates to health crises and rapid training on pharmacological features. This information can be helpful in future situations where there is a lack of knowledge about a new pathology and where pharmacists can provide therapeutic advice. Moreover, knowing that a higher number of DRP have been reported in patients with COVID-19, tools like a PWS system can help clinical pharmacists and physicians to prevent, identify and solve such issues efficiently.

## Data Availability

The original contributions presented in the study are included in the article/supplementary material, further inquiries can be directed to the corresponding author.
